# Mr. Brown, on the Necessity of Establishing an Hospital for the Treatment of Ulcerated Legs

**Published:** 1800-02

**Authors:** Charles Brown

**Affiliations:** Hatton Garden, No. 25


### Mr. Brown, *on the Neceſſity of eſtabliſhing an Hoſpital for the Treatment of Ulcerated Legs.*

---

### *To the Editors of the Medical and Phyſical Journal.*

Gentlemen,

Among the various charities which do honour to this city, there ſtill remains *one* wanting, which I am certain deſerves as much the attention of the humane as any yet eſtabliſhed; I mean, an hoſpital, infirmary, or diſpenſary, for the ſole reception of ulcerated legs. It is a very melancholy fact, that among the lower claſſes of the community, nearly in the proportion of one out of five, labour, and have many years, under this ſevere affliction, which to them is a conſtant ſource of expence, and muſt ultimately terminate their exiſtence, by ſubjecting them to other diſorders more fatal and rapid in their progreſs. It will, perhaps, be urged as an argument againſt putting ſuch a benevolent ſcheme into execution, that our hoſpitals, as, St. Thomas's, Guy's, St. Bartholomew's, the London, don, and all others, indeed, are open for the reception of ſuch objećts, and therefore there is no abſolute neceſſity for eſtabliſhing one ſolely for this end. But when we pauſe for a moment, and reflećt, that many labouring under this loathſome diſeaſe, do apply at theſe hoſpitals, and are denied admiſſion, and that many others are turned out incurable, who, under the ſkilful care of ſome ſurgeon, (who, perhaps, devotes more of his attention, and ſupplies the patient with thoſe neceſſaries which an hoſpital cannot, or does not furniſh,) receives a ſpeedy and effećtual cure, ſurely no one but who is inſenſible to the agonies of the mind, and painful diſorders of the body, will refuſe his mite. From great and (with diffidence I affirm it,) very ſucceſsful praćtice in this branch of ſurgery, I can aſſert, that among the many caſes of ulcerated legs, which I have had under my care, ſince I have been in praćtice in this metropolis, I have never found any difficulty in effećting cures, even in the very worſt caſes, by attention to the particular circumſtances of the ſtate of the ulcers, by cleanlineſs, and occaſional reſt. It would require but little expence to put this charity on a footing with the moſt reſpećtable in London. About £.2000 would erećt a building ſufficiently large for the purpoſe, and a ſubſequent fund of about £.500 annually would ſupport it. The remedies required for healing bad legs, are very few and cheap, and ten thouſand perſons, yearly, might receive benefit from this charity. It only requires ſome ſpirited perſons to ſtep forward, and intereſt themſelves warmly in behalf of ſome hundred thouſands, ſorely afflićted with the diſorder, who would offer up their prayers for their benefaćtors; and from the ſtate of poverty they are now in, would be enabled to enjoy ſome of the few comforts of life. If any perſons are willing to aſſiſt the writer of this, in attaining an objećt ſo deſirable, and who is ready to ſubſcribe fifty pounds towards the fund, they will be ſhewn a plan, drawn up for the purpoſe, and requiring only the ſum before ſpecified to ſet it on foot.

I have the honour to be,

Your obliged,

CHARLES BROWN.

*Hatton Garden*, No. 25,

*Nov.* 5, 1799.

